# ‘Climate change concerns human survival…and justice in our international community’: A corpus-based positive discourse analysis (PDA) of the largest developing nation’s global involve/engagement discourses (re)told in interpreting

**DOI:** 10.1371/journal.pone.0277705

**Published:** 2023-04-20

**Authors:** Chonglong Gu

**Affiliations:** The Hong Kong Polytechnic University, Hong Kong, Hong Kong; University of Almeria, SPAIN

## Abstract

Contributing to a much-needed ‘outward turn’ in interpreting studies, this intervention examines the role of interpreting and interpreters in (re)articulating the welcome ‘voice’ of a developing nation in the global South. Against the backdrop of reform and opening-up (ROU), China, the world’s largest developing country, is increasingly open and keen to engage globally. Such elements as openness, integration, and international engagement represent vital components of the overarching ROU metadiscourse that justifies China’s sociopolitical system and multifarious policies and decisions. As part of a series of digital humanities (DH) informed empirical studies exploring the part played by interpreting in rendering China’s ROU metadiscourse, this study zooms in on the government interpreters’ mediation of Beijing’s international engagement and global involvement discourses. Unlike CDA which often foregrounds the negative themes (e.g. injustice, oppression, dominance, and hegemony), an innovative corpus-based positive discourse analysis (PDA) is introduced and applied, drawing on 20 years of China’s press conferences. This article points to the interpreters’ visibility and agency in facilitating and strengthening China’s discourse through (over)producing core lexical items and salient collocational patterns. Following the trends of interdisciplinarity and digital humanities, this corpus-based PDA study illustrates ultimately how a major non-Western developing country from the global South communicates its discourse bilingually in front of the international community. The potential impact and implications of the interpreter-in(tro)duced discursive changes are discussed *vis-à-vis* the ever shifting and delicate East-West power balance from the perspective of (geo)politics

## 1. Introduction

China’s rise represents one of the most important single events in our time. Partly as a result of the pragmatist Reform and Opening-up (ROU) programme that began in 1978 under Deng Xiaoping, China has registered decades of steady economic growth and is on track to becoming the world’s largest economy [1]. China’s meteoric rise [2] may be welcomed to varying extents globally by different countries, which may also give rise to civilisational clashes and geopolitical tensions. However, geopolitics, ideology and rhetoric aside, China’s recent development is nothing short of an impressive success story in terms of how a non-Western country from the Global South can successfully develop and transform itself on various fronts. Moving beyond the more centralised planned economy and relative isolation witnessed in the period between 1949 and 1978, China under the Reform and Opening-up policy has decided to let the market play a bigger role, pursuing a ‘socialist market economy’ and exploring a path of ‘socialism with Chinese characteristics’. This period of ROU features great openness, flexibility and a bold can-do attitude, which more or less have remained the order of the day since then. The pragmatism and openness in pursuing market-oriented reforms are evidenced in Deng Xiaoping’s explorative and open-minded learn-as-you-go approach of “crossing the river by feeling the stones”. Also, China’s openness and willingness to learn from other systems and good practices are epitomised in his widely quoted metaphor that “a cat is a good cat if it catches mice, whether black or white”.

The ROU, as such, is a vital watershed in China’s recent history, witnessing the country’s rapid development and increasing interaction with the rest of the world. Indeed, like Enlightenment, the clash of civilisations, environmentalism, modernity, progress, the war on terror, multiculturalism, Black Lives Matter, Capitalism, ROU can be viewed as an all-encompassing metadiscourse or meta-narrative (French: *grand récit* or *métarécit*). Containing various micro-discourses, a broader metadiscourse or meta-narrative accrues and can enjoy considerable currency over long stretches of time [3–5]. In fact, the overarching ROU metadiscourse [6] permeates in different aspects of China’s sociopolitical and economic developments and day-to-day operation and legitimatises China’s political and economic systems, style of governance, decision-making, policies and initiatives, and its diplomatic stances and positions in the entire post-1978 period. ROU at the core also represents an exhilarating development discourse, showcasing the priorities, experiences and even thinking patterns of a major developing country. Given the obvious international dimensions and partly the outward-facing nature of the ROU, how the ROU metadiscourse is conveyed and communicated may well have far-reaching ramifications and consequences globally (e.g. contributing to power shifts between the global South and North).

Despite the clear discursive element involved, the influential ROU has so far mostly been investigated from the standpoints of economy, business, development studies, and the social and political sciences [1, 7, 8]. As such, there has been a lack of sufficient scholarly engagement with how the ROU discourse has been constructed and communicated. In particular, there has been even less attention so far in terms of the (re)construction of the ROU metadiscourse bilingually in translation and interpreting, barring two recent studies [6, 9]. In translation and interpreting studies (TIS) in general, drawing on a (critical) discourse analytical or a social narrative approach, there has relatively recently been growing recognition in terms of the crucial agency and mediation role of translators [3, 4, 10, 11] and interpreters [12–14] from various conflictual, sociopolitical, institutional and historical contexts via more manual qualitative analysis. Over the recent few years, benefitting from what is hailed as a ‘useful methodological synergy’ [15], corpus-based (critical) discourse analysis [16–20] has been increasingly incorporated to enrich the otherwise manual and textually-oriented research and to deliver more systematic and objective analysis of the translation and interpreting products. This makes it interesting to adopt a bilingual discursive communication and interpreting studies perspective and explore how the ROU discourse is mediated and (re)constructed in interpreting drawing on corpus linguistics.

Indeed, in addition to such common topics as ‘reform’, ‘economy’, ‘development’ and ‘market’, the international involvement and global engagement discourses represent essential components and recurring themes of the broader reform and opening-up (ROU) metadiscourse. The international involvement and global engagement discourses, as such, will form the focus of this corpus-based study as useful entry points into the broader ROU metadiscourse. Given the relatively neutral/positive nature of the themes (which constitute the development discourse of a major non-Western developing country), an innovative corpus-based positive discourse analysis (PDA) is carried out to (de)construct and explore its discursive articulations (see section 3 for more discussions on the framework and methodological approach adopted). Framed within the wider contexts of interdisciplinary research and digital humanities (DH), the pragmatist and triangulatory mixed-methods approach of corpus-based PDA is used on a large corpus containing China’s Premier-Meets-the-Press conference data from 1998 to 2017 (20 years) so as to establish the government-affiliated interpreters’ potential agency and discursive mediation in (re)constructing China’s discourse. Viewing interpreters as major sociopolitical actors, this study explores their potentially vital role from a broader international communication and sociopolitical perspective, beyond the traditionally narrow conceptualisation of interpreting as a relatively mechanical process within a self-contained and semi-closed system. This study is one of the first studies to draw on corpus-based PDA and arguably the first one to adopt this approach in translation and interpreting studies. This empirical study, interdisciplinary in nature, promises to contribute *inter alia* to scholarship relating to translation and interpreting, bilingual communication, discourse studies, corpus linguistics, and the social and political sciences.

## 2. China’s reform and opening-up metadiscourse as ‘development discourse’ of the global South

Development discourse relates to the discursive articulations on development most notably in relatively poor and disadvantaged developing or third world countries in the Global South (e.g. Brazil, Indonesia, Nigeria, Egypt, Cuba, China, Bangladesh, India, South Africa, and Pakistan). It is concerned with the sociopolitical, economic, infrastructure, health, and education developments in (often postcolonial) societies in Africa, Asia, Oceania, and Latin America. The development of these countries is for example explored extensively in the interdisciplinary area of development studies. In our multi-polar world in the 21^st^ century, the developing world in the Global South is becoming a force to be reckoned with, which is poised to play an increasingly bigger role in the international community. Notably, the dynamic mutually enriching exchanges within the BRICS (Brazil, Russia, India, China and South Africa) countries represent a fine example of South-South cooperation, aiming to advance the development interests of a group of rapidly developing and emerging nations in a similar stage of development [21].

As the world’s largest developing country, China’s development discourse taken from its own ROU experience is no doubt one of the most consequential and telling. In contemporary China, there are two major overarching discourse modes, that is, a revolutionary discourse (1949–1977) and a reform and development discourse (1978—now). The first period involved a few (eventful) decades of close-door socialist experimentation since 1949. In view of the relative isolation, backwardness and poverty witnessed in this period, China under Deng Xiaoping has opted for a path of “Socialism with Chinese Characteristics” since 1978. This represents a major policy and discursive shift towards a more practical and outward-looking approach that focuses on reform, economic development and better integration internationally. Deng’s ROU, as such, represents a pragmatic and flexible *modus vivendi* incorporating elements of market economy and capitalism to China’s overall socialist system featuring a degree of macro-control from the state [22].

China’s ROU, *inter alia*, is about such crucial aspects as economy, market, opening-up, reform, and international engagement. However, it is fundamentally about concrete developments and modernising the country on various fronts. As an example of the overriding focus on development in the post-1978 period, Deng Xiaoping remarked that “development is the only hard truth” (*fazhan caishi yingdaoli*) and China must develop itself in various aspects. China’s ROU metadiscourse itself represents part of a wider development discourse in the global south [9], encapsulating various objectives and plans, concrete practices and policies, useful experiences, as well as developmental views, path, philosophy and wisdom unique to the Chinese context. Recently, as China develops economically and geopolitically, the country is also seeking to boost its discursive power and have its voice heard globally. This is evidenced clearly in the official slogan *jianghao zhongguo gushi* (to tell the Chinese story properly) in recent years. Against such a backdrop, China’s ROU metadiscourse, as the development discourse of the world’s largest developing country, when interpreted into English, can have wider ramifications and may effect change to the ever-shifting power differentials between the North and South and the East and West in the global arena discursively and beyond.

## 3. Theoretical framework and methodology: Corpus-based positive discourse analysis as part of interdisciplinary digital humanities research

Our recent technological developments have afforded researchers the ability to carry out computer-powered digital research into the different areas of humanities and social sciences. This has given rise to the exponential expansion of digital social sciences and digital humanities over the last decade or so, forging increasing cross-fertilisation between different fields. Framed within a broader context of digital humanities and interdisciplinary research, the mixed-methods approach of corpus-based PDA is adopted in this article concerning interpreting studies, bearing the nature of the study in mind.

### 3.1 Positive discourse analysis

Discourse can be viewed as a form of *social practice that is both socially conditioned and socially shaping*. *Traditionally*, *critical discourse analysis (CDA)*, *in its various manifestations*, *has been a major ‘go-to’ framework to investigating the ideology and power* reproduced and legitimised through discursive means [23, 24]. However, while the aim is not necessarily to criticise, the more dominant and established CDA tends to engage more with the hidden and asymmetrical power relations and the often opaque and sometimes oppressive, sinister and insidious ideologies enacted in discourse. That is, although CDA might already include a small degree of engagement with the ‘positive’ elements [25] in discourse, arguably the major themes explored in the traditional CDA remain to be negative ones including injustice, dominance, power asymmetry, hegemony and control.

However, in reality, as with anything, discourse is not always negative, oppressive, exploitative and sinister in nature. Discourse, despite the ideological and socially shaping nature, can also be progressive, empowering, emancipatory, liberating, and morale-boosting and serve as a form of positive resistance to right the wrong and level the playing field etc. Although it is not always easy to definitively say what is positive or negative, discourses in various scenarios might be considered to be positive and progressive in terms of the underlying intention and/or the discursive effect. Examples of such positive discourses can be the powerful discourse against racism (e.g. during the Black Lives Matter movement), the unifying discourse of solidarity against violence and terrorism, the emancipatory discourse against colonialism in previously colonialised societies, the discourse that helps to lower the temperature and extend the olive branch for peaceful dialogue and harmonious co-existence (e.g. the discourses articulated in the lead-up to and during the historical meeting between Kim Jong-un and Donald Trump in Singapore in 2018), the positive and encouraging development discourse of the global south that provides an alternative voice and challenges the more dominant ‘North’ (e.g. the broader reform and opening-up discourse from China).

Despite the existence of more positive discourses, there has been a lack of sufficient scholarly engagement with the neutral/positive elements in discourse analysis. There have indeed been growing calls for CDA to move beyond the almost exclusive focus on the negative aspects [26] and be more attentive to the progressive and positive elements instead. In response to this, Positive Discourse Analysis (PDA) was proposed by James Martin [27], which has also been discussed by a few scholars [28, 29]. Unlike CDA that focuses on the more negative aspects of discursive construction, PDA has a different agenda. As an advocate for PDA, Martin [27] discusses convincingly the justifications for a useful move towards PDA as a natural and vital step beyond and a useful complement to the traditional preoccupations of CDA. It is worth noting that PDA itself is firmly embedded within the existing work of CDA and indeed has significant overlap with CDA. However, it goes one step further to (de)construct how discourse might be used for articulating an alternative voice [28], effecting more positive change we would like to see, and ultimately making our world a more equal and better place. As such, this highlights the need to reclaim, valorize, and empower resistant discourses from the non-Western and even third/fourth world. Despite the differences in agenda, PDA and CDA are the *yin* and *yang* [27] that contribute to a broader approach of critical discourse studies, which ultimately aims to (de)construct ideology in discourse.

Compared with CDA, PDA is relatively new and remains less explored and even ‘marginal’ [29] in places such as the US. Of the relatively very small body of work relating to PDA, qualitative manual analysis is predominantly applied. Focusing on the Chinese context, Su [30] used PDA and explored Chinese president Xi Jinping’s political speech delivered in Singapore. Also, framed within a context of anti-colonialism and anti-imperialism efforts, Nartey [31] looked at how the progressive and resistant discourses are constructed in Ghana’s independence leader Kwame Nkrumah’s address.

In the UK setting, drawing on PDA, Sauntson [32] devoted part of the analysis to unpacking the positive discourse of resistance against the discriminatory discourse used by the people who are anti-LGBTQ+. Also, Bartlett [28] used the framework in the Guyana context, arguing that using an ethnographic method might potentially further enhancing PDA by placing the discursive product within a wider context in terms of the customs and routines of the social actors producing it. Also, Agustín [33] employed the PDA framework and investigated the ways in which Danish and Spanish non-governmental organisations have managed to forge understanding and empathy with refugees. Notably, most of those PDA studies were conducted in a manual and qualitative way based on small datasets.

So far, very few PDA studies, if any, have been carried out in translation and interpreting (let alone in the Chinese setting). The reason why positive discourse analysis is applied in this study as opposed to the traditional CDA is because the discursive articulations of a developing nation from the global South on more open-minded international engagement and global integration, while not necessarily immune from ideological considerations, are essentially positive or at least neutral in nature. This is particularly true when compared with, for instance, the negative isolationist discourses calling for (self)isolation and withdrawal from international affairs and global involvement (e.g. the anti-immigrant rhetoric and anti-globalism and anti-globalisation ‘America First’ discourse in the Donald Trump administration in the US). As a positive/neutral discourse [27, 31], the discursive articulations of openness and global engagement under discussion in this study presumably can serve to maintain the good momentum of development domestically and help win the hearts and minds of people in the developing world and beyond. As such, PDA serves as a suitable framework for this study.

### 3.2 Corpus-based PDA: A powerful mixed-methods marriage

Similar to the common criticisms the traditionally qualitative CDA is subject to relating to the lack of objectivity and systematicity and the possibility of cherry-picking on the part of researchers [34], the mostly manual PDA faces similar accusations. For more objective and comprehensive analysis and valid findings, methods of corpus linguistics (CL) have been increasingly utilised alongside CDA *as* a “useful methodological synergy” [15, 35]. As demonstrated by Bednarek and Carr [36], a CL approach represents a highly powerful method in digital social science and digital humanities research. For them, CL is well positioned to offer relatively systematic insights into the data at various levels yet is significantly less demanding and requires less specialised expertise on the part of scholars compared with other more sophisticated methods requiring technical know-how (e.g. programming). So far, a digital humanities approach (e.g. using corpus linguistics) has been applied in a limited yet growing body of research. These include monolingual (critical) discourse analysis [15], literary translation [37], and interpreter-mediated political communication [6, 9, 16] in different contexts and settings.

As such, framed within the broader context of interdisciplinary research and digital humanities (DH), the mixed-methods approach of corpus-based positive discourse analysis (CBPDA) is employed in this study to triangulate between the typically qualitative (PDA) and the typically quantitative (CL) for more systematic and thus more convincing findings. Despite the fact that interpreting as a form of bilingual discursive communication can also effect (positive) change on a broader socio-political level, this framework is rarely explored, if at all, in interpreting studies. This article is one of the first studies in interpreting to draw on PDA and arguably the first study to use a corpus-based PDA approach in studying bilingual discursive communication.

## 4. Corpus data

The corpus-based PDA analysis is carried out on the CE-PolitDisCorp (Chinese-English Political Discourses Corpus) established to explore the different aspects of political interpreting between Chinese and English. Containing 310,924 tokens (170,260 tokens in Chinese and 140,664 tokens in English), the CE-PolitDisCorp represents a relatively large interpreting corpus, featuring 20 years of China’s Premier-Meets-the-Press conference data (1998–2017). The interpreter-mediated and televised premier’s press conference was itself established against a backdrop of greater international engagement as part of China’s Reform and Opening-up efforts. That is, the conferences gradually became routinised as a high-profile annual discursive event [38] for better communication globally. The press conferences feature questions asked by domestic (e.g. from CCTV and People’s Daily) and foreign journalists (e.g. from BBC, CNN, and NBC) on a whole gamut of social, economic, political, and diplomatic topics (e.g. China’s economic growth, China’s infrastructure development, Covid-19, China-US relations, China-Russia friendship, trade frictions, Hong Kong, and Taiwan). One press conference, on average, lasts for about 2 hours and there are 20 such conferences included in the corpus data, covering 3 recent government administrations: Jiang Zemin-Zhu Rongji (1998–2002), Hu Jintao-Wen Jiabao (2003–2012) and Xi Jinping-Li Keqiang (2013–2017). Given the extended nature of the data, it is interesting to gain useful diachronic perspectives and identify relatively consistent patterns and statistical information over a span of two decades. It thus represents an invaluable source for this empirical study as the corpus contains data concerning discourses about China’s openness and global engagement (e.g. entry into the WTO, contributing to global economy during economic recession, and foreign investment). The data was transcribed *verbatim*. After careful checking and preparation, the corpus-based PDA is carried out, using the AntConc software developed by Laurence Anthony. A detailed breakdown of the corpus data can be found in Table 1. For more information on the corpus data itself, please see Gu [16, 17].

**Table 1:** The CE-PolitDisCorp corpus.

Subcorpus A	Subcorpus B	Subcorpus C (subcorpus A+ subcorpus B)	Subcorpus D
China’s official discourse in Chinese	China’s interpreted discourse in English	China’s official discourses in Chinese and English	Journalists’ questions and their respective interpretations
127,696 tokens	105,495 tokens	233,191 tokens	77,733 tokens

Since the bulk of the corpus data concerns (1) Subcorpus A (the Chinese premiers’ answers to journalists’ questions in Chinese) and (2) Subcorpus B (the corresponding content interpreted into English), these two components will represent the sole focus of this article. The interpreters involved are often communist party members and civil servants affiliated with the foreign ministry of the Chinese government. Those high-profile conferences are consecutively interpreted and broadcast live.

## 5. Data analysis

**Fig 1:**
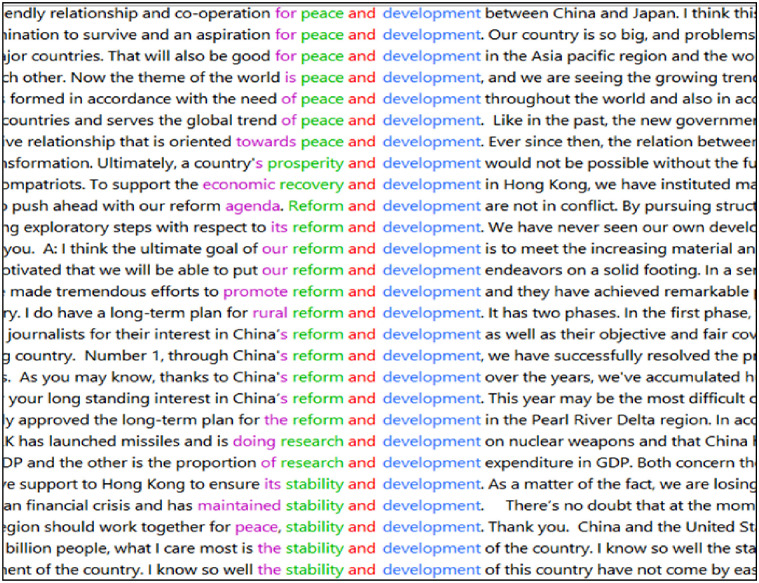
Collocations relating to ‘development’.

The interpreting of China’s international engagement and global involvement discourse can be explored from different angles and through examining different lexical items. For example, as illustrated in Fig 1, searching ‘development’ in the corpus data and sorting to the left can reveal interesting patterns (e.g. ‘peace and development’ and ‘stability and development’), which show that cognitively China highly appreciates a peaceful and stable international environment in its global engagement as a developing nation. However, given the limited space, attention is focused firstly on the interpreters’ rendering of key concepts of China’s broader ROU metadiscourse at an overall lexical level to offer a general idea of the interpreters’ mediation (statistically, the more times certain lexical items belonging to one particular discourse are (re)produced by the interpreters, the discourse itself is made more prominent and emphatic).

Attention then will focus specifically on **items semantically related to the globe or being international** as a proxy or entry points to the data. In terms of the specific criteria, relevant items are selected from the frequency list (cf. Table 2 for more details). The interpreters’ mediation will be examined at lexical, diachronic and collocational levels. Since the more data-driven CL methods are incorporated to guide my analysis, the PDA framework will be operationalised in a more generic manner as a source of theoretical insights (without following any *a priori* linguistic categories).

**Table 2:** Frequencies of items related to ROU at an overall level in Chinese and English.

Broader concept/discourse	Chinese subcorpus (freq)	English subcorpus (freq)	ST-TT Change (percent)
INTERNATIONAL ENGAGEMENT AND GLOBAL INVOLVEMENT	全球*/世界*/国际*(302)	glob*(including global, globalize, globalized, globalization)/world*/international* (including international and internationally) (362)	+19.87
DEVELOPMENT	发展* (451)	develop* (539)	+19.5
ECONOMY	经济* (450)	econom* (526)	+16.9
REFORM	改革* (323)	reform*/restructur* (392)	+21.4
MARKET	市场* (172)	market (227)	+32
OPENNESS	开放/放开/敞开/公开 (121)	open* (136)	+12.4
MODERNIZATION	现代* (44)	modern* (45)	+2.3
SOCIALISM	社会主义* (42)	socialis* (43)	+2.3
HARMONY	和谐* (11)	harmon* (including harmony and harmonious) (15)	+36.3
STABILITY	稳* (including 稳定, 稳定性,稳健, 稳固, 平稳 etc.) (157)	steady, stable, stabili* (including stability, stabilize, stabilized, stabilizing, stabilization) (156)	-0.64
Overall (freq)	2,073	2,441	+17.75

### 5.1 Interpreters’ overall mediation on constitutive concepts and themes relating to ROU

Since the overarching Reform and Opening-up metadiscourse is inevitably realised in various main themes or discourses, DEVELOPMENT, ECONOMY, REFORM, INTERNATIONAL ENGAGEMENT AND GLOBAL INVOLVEMENT, MARKET, STABILITY, OPENNESS, MODERNISATION, SOCIALISM, and HARMONY have been identified as of particular relevance. The identification of those salient themes is both based on lexical frequency in English and the researcher’s expertise in the related topics. The various concepts and themes’ instances in both languages are presented in Table 2. To retrieve and count the different (lexical) realisations within each theme/discourse, the wildcard function (*) was used.

This overall analysis at a lexical level reveals that core concepts and themes constitutive of the ROU metadiscourse have been rendered significantly more prominent by the government-affiliated interpreters in English *vis-a-vis* the Chinese original over a span of 20 years (a 17.75% increase overall and noticeable increases in all thematic categories except one category). Such a focus on lexical items, however crude, is vital discursively. This is because the Chinese premiers’ frequent mentioning of certain important items (e.g. harmony, reform, market, economy and openness) indicates both discursively and cognitively how important certain aspects are for China’s reform. For China experts and the media alike, how many times certain keywords are mentioned is routinely used as a useful indication of China’s shifting policies, preoccupations, and priorities [6].

The interpreters’ proliferated (over)production of relevant lexical items in the decision-making process represents an interesting case of repetition [6, 23] and is ideologically salient. Discursively and rhetorically, if an (under)production of relevant lexical items in interpreting leads to the ROU metadiscourse being diluted and undermined, then such noticeable (over)production suggests a strengthening of the Chinese source discourse and a reinforcement of the government’s institutional ideology in English, thus indicating a strong level of institutional alignment with the government’s official policies and positions on the part of the interpreters. Framed within a broader context, where China is seeking increasing discursive power globally and wants to have the Chinese story properly told (*jianghao zhongguo gushi*), the interpreters play a vital role in (re)telling China’s reform and opening-up story emphatically overall. Regarding the discursive effect and broader implications, the interpreters’ emphatic rendering of China’s ROU metadiscourse constitutes an alternative voice, which discursively might serve to counterbalance the often naturalised, dominant, and taken-for-granted narratives [39] from those developed nations in the West [40]. Given the outward-facing, televised, and consequential nature of the communicative event, this might have far-reaching impact and can effect change to the existing power differentials between the East and West and the South and North at least discursively. As far as image is concerned, the interpreters’ discursive mediation has (re)constructed a more favourable image of the government being highly development-focused, reform-minded, keen on modernisation, and increasingly engaged in the global arena and a positive image that China is well on track in its development efforts (cf. [6] for more details on the discursive effect of this overall mediation).

### 5.2 Interpreters’ mediation on China’s global involvement and international engagement discourses

Having discussed the interpreters’ general tendency to (over)produce lexical items, more focused analysis is conducted on the interpreters’ (re)presentation of China’s (positive) discourses relating to its global involvement and engagement, which directly indicate China’s openness and willingness to interact internationally. The discussion will be presented at a lexical, diachronic and collocational level. Bilingual examples of PDA will also be provided.

#### 5.2.1 Interpreters’ overall level of mediation on China’s global involvement and international engagement discourses

Firstly, for an overall idea, in both subcorpora, 全球* and glob* (globe, global, globalise, globalised, globalising, globalisation, globally), 世界* and world* (world and worldwide *etc*.) and 国际* and international* (international and internationally) were searched. Some of the representative concordance lines indicative of China’s global engagement and international involvement are: ‘China takes an active part and safeguards the international system’, ‘we are willing to work with the countries of the world to maintain global peace’, ‘China will shoulder our due international obligations as a large developing country’, ‘China will continue to work with other countries to advance the international efforts in tackling climate change’, ‘China’s economy is already tied to the globalised economy’, ‘China is ready to work with other countries to further improve the international governing system’, and ‘China is ready to work with other countries in the world in building a fair and equitable new international political and economic order’. These show China’s keen interest in and commitment to interaction globally as a developing country and indicate various positive actions (e.g. such verbs as ‘safeguard’, ‘work’, ‘shoulder’, ‘advance’, ‘improve’, and ‘build’).

Statistically speaking, in the corpus, there are 16 mentions of 全球* and 60 mentions of glob* (a 275% increase), 165 mentions of 世界* and 158 mentions of world* (a 4.2% decrease) as well as 121 mentions of 国际* and 144 mentions of international* (a 19% increase). Therefore, while there are 302 instances of lexical items concerning the globe, world and the international in Chinese, there are 362 instances of their counterparts in English. This represents a noticeable 19.87% increase, thus pointing to the government interpreters’ increased institutional alignment. At least discursively, this (re)constructs a positive image in English that China is even more open and global minded in front of the international audience.

Diachronically, the premiers’ discourse in Chinese appears to be growingly internationally minded and globally oriented over the three administrations (increasing from on average 8 to 16 and 20.4 times per year), as seen in Table 3. This seemingly shows that, over time, China as a developing country has shifted progressively from a relatively closed, isolated and inward-looking country to one that is more globally oriented and proactive in international affairs. This diachronic discursive trend identified is also evidenced in major events including China’s entry into the WTO, its founding of the Asian Infrastructure Investment Bank (AIIB) and the initiation of the ‘Belt and Road Initiative’. Recently, China also talked about ‘China solutions’ to addressing some of the world’s pressing issues and called for a new type of major-country relationship with the current established power, that is, the United States. The government interpreters have clearly exhibited increased institutional alignment with the government in all of the three administrations, discursively further strengthening and facilitating China’s international involvement and global integration discourses in English. The government interpreters’ (repeated) additions of items and expressions relating to the world and the globe or efforts to make related items more explicit and apparent can be found in examples below (the underlined sections are added or made more explicit and emphatic by the interpreters).

**Table 3:** Lexical items relating to the world and globe in both subcorpora and diachronically across the administrations.

	Chinese subcorpus (freq/freq per year)	English subcorpus (freq/freq per year)	Increase (percent)
Zhu (1998–2002)	40/8 (7082.6 tokens/year)	48/9.6 (6101.6 tokens/year)	20%
Wen (2003–2012)	160/16 (8614.5 tokens/year)	198/19.8 (7166.3 tokens/year)	23.75%
Li (2013–2017)	102/20.4 (9740.4 tokens/year)	116/23.2 (7698.6 tokens/year)	13.73%
Overall	302	362	19.87%

**Example 1 (2010):**
**ST**: 我真诚希望美欧承认中国的市场经济地位, 并且放开高科技产品对中国的出口, 这有利于贸易的平衡。
**Gloss**: I sincerely hope that the US and Europe will recognize China’s market economy status and open up their export of high-tech products to China. This is beneficial to trade balance.
**TT**: I sincerely hope that Europe and the United States will recognize China’s market economy status and lift restrictions on the exports of high technology commodities to China, because that will help promote trade balance in the world.
**Example 2 (2017):**
**ST:** 提高中国产品的质量, 迈向中高端, 必然要更大地打开大门, 更多地引进国外先进的技术和产品。
**Gloss:** To improve the quality of China’s products and to enter into the medium and high end (market) necessitate opening the door wider and introducing advanced foreign technologies and products more.
**TT:** The requirement for raising our own products’ quality and upgrading our own industries to a medium-high level actually needs us to open even wider to the outside world by introducing more advanced products and technologies.

Although seemingly insignificant individually, taken together, the interpreters’ such repeated additions and more explicit mentions of items relating to the world or the globe in English cumulatively help (re)construct a favourable image that China is even more global minded and internationally oriented in English compared with the Chinese STs.

#### 5.2.2 Collocational patterns concerning China’s international involvement and global engagement

Beyond an overall lexical level, more focused discussions are provided on international* and global* as well as their corresponding collocational patterns here. An exploration of collocations is useful because discourse is inevitably constructed through collocations or the ‘company’ [41] certain lexical items keep and a word’s collocations often represent ‘statements of the habitual and customary places of that word’ [41].

*5*.*2*.*2*.1 International*. International* was firstly searched in the English subcorpus to retrieve concordance lines, which were then sorted to the right and left to identify patterns. Some of the identified patterns are: *international financial crisis* (22), *international community* (9), *international market*(*s*) (8), *international environment* (8), *international practice*(*s*) (6), *international issues* (3), *international law*(*s*) (3), *international obligations* (3), *international currency* (3), *international effort*(*s*) (2), *international organisation*(*s*) (2), *international standard*(*s*) (2), *international asset* (1), *international commodity prices* (1), *international trade* (1), *international resources* (1) and *international rescue effort* (1). These patterns are mostly accurate (re)presentations of the premiers’ utterances after a comparative analysis with the Chinese STs. Yet, ‘international community’ and ‘international practice(s)’ prove to be two salient patterns. More detailed discussions can be found below.

**(a) International community**

‘International community’ is defined by the Oxford Dictionary as ‘the countries of the world considered collectively’. Often used by politicians and journalists in the realms of geopolitics and international relations, the term conveys a sense of legitimacy and credibility through the indication of common consensus worldwide on certain issues. The term is not without its critics. Noam Chomsky argues that the inclusive sounding ‘international community’ is used almost exclusively to refer to the United States and its Western allies. Similarly, Martin Jacques sees the term as a way of dignifying the west, of globalising it, of making it sound more respectable, more neutral and high-faluting. The term, as Wang’s [42] corpus-based analysis suggests, can assume different meanings for different justifying purposes.

**Fig 2:**
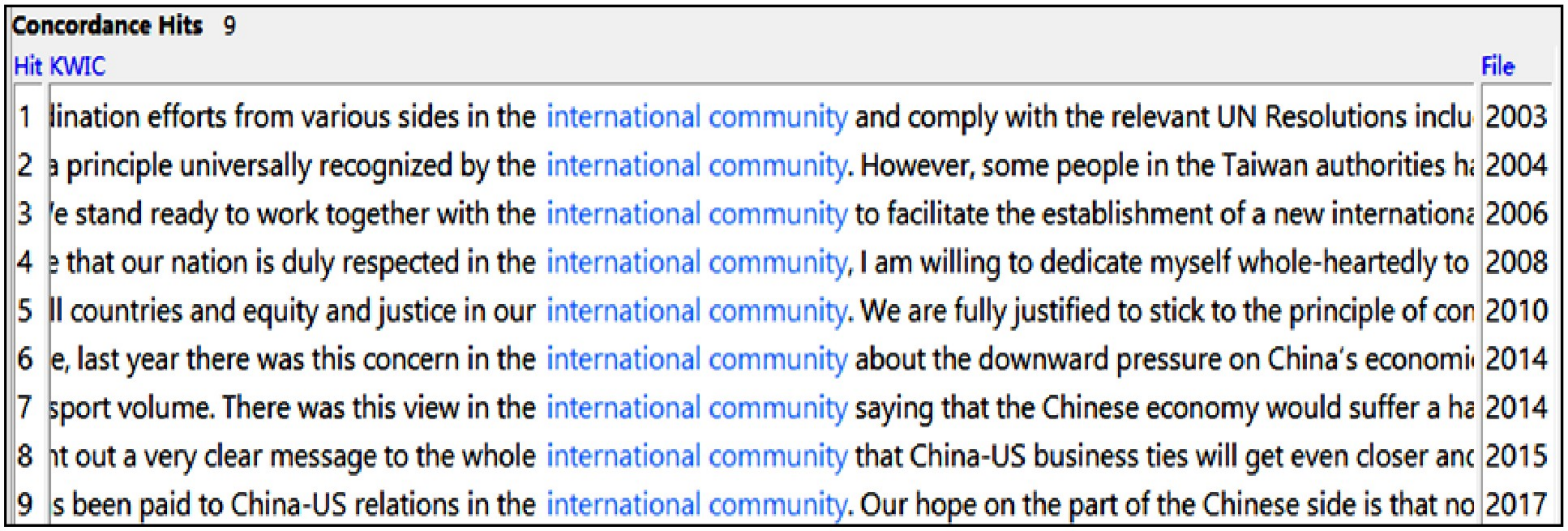
Screenshot of concordance lines featuring ‘international community’ (by year).

An investigation in both subcorpora shows that there are 9 instances of ‘international community’ in English (see Fig 2) yet only 3 instances of its literal equivalent国际社会 in Chinese (a 200% increase). Comparative analysis in subcorpus C reveals that, of the 9 instances of ‘international community’, only 2 are triggered directly by the ST (22.2%), 6 are triggered indirectly by ordinary and unmarked items semantically related to the ‘world’ (国际, 世界, 世界, 国际上, 世界, 全世界) and 1 is not even triggered by the ST but added by the interpreter.

The interpreters’ proliferated use of ‘international community’ in English is salient ideologically, representing a strengthening of the Chinese originals. The functions of the interpreters’ employment of the term can be categorised into the following aspects:

(1) *To lend further credence and legitimacy to China’s (territorial) claims*:

This is evidenced in the example below (‘international community’ is triggered by the ST in this case), which suggests that China’s claim is justified and universally recognised and supported. Such usage is similar to that used by Western countries to show universal acceptance or recognition.

**Example 3 (2004)**
**ST**: There is but one China in the world. Both the mainland and Taiwan are part of China. The sovereignty and territorial integrity of China allow no division. China’s sovereignty over Taiwan has been explicitly recognised in the Cairo Declaration and the Potsdam Proclamation. And it is a principle universally recognised by the international community.
(2) *To translate general items relating to ‘the world’ as ‘international community’*:

Discursively, compared with using the more generic items related to the ‘world’, the interpreters’ repeated mentions of the family-like conceptualisation ‘international community’ emphasise a strong sense of togetherness and international membership, thus helping portray China as a relevant, important and visible member of a big family of nations in English. This is illustrated in the following examples in English.

China’s expected position in the world: ‘to ensure that our nation is duly respected in the international community’ (2008); the discourse of Sino-US ties: ‘a great deal of attention has been paid to China-US relations in the international community’ (2017) and ‘it has already sent out a very clear message to the whole international community that China-US business ties will get even closer’ (2015); Also, China’s climate change discourse: ‘the issue of climate change concerns human survival, the interests of all countries, and equity and justice in our international community’ (2010).

The mentions of the more emphatic ‘international community’ in these examples are respectively triggered by general unmarked items 世界 (the world), 全世界 (the whole world), 世界 (the world) and 世界 (the world) in Chinese. As such, interpreters’ repeated use of the expression represents a small degree of strengthening compared with the Chinese STs.

(3) *The interpreter’s addition of ‘international community’ untriggered by the ST*.

Another scenario involves the interpreters’ addition of ‘international community’ that is not triggered by the ST at all. The addition therefore helps portray China as an active member and important stakeholder of a bigger interconnected family of nations in an emphatic manner. This is exemplified in: ‘Last year there was this concern in the international community about the downward pressure on China’s economic growth’ (2014).

To sum up, through the repeated use of ‘international community’, discursively, the interpreters have aligned institutionally and helped further boost China’s claims, emphasise China’s international membership and facilitate its global integration in the English discourse.

**(b) International practice(s)**

**Fig 3:**
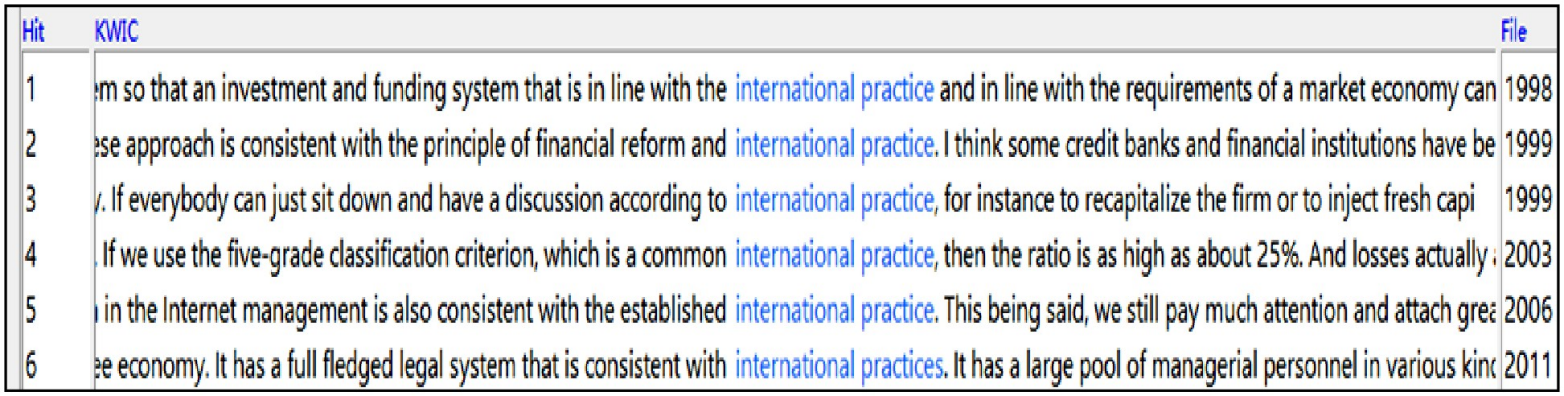
Screenshot of concordance lines featuring ‘international practice(s)’ (by year).

Having discussed the collocation ‘international community’, attention is now focused on another interesting pattern ‘international practice(s)’. Statistically, there are 6 instances of ‘international practice(s)’ in the English subcorpus (Fig 3), which are associated with ‘in line with’ (line 1), ‘consistent with’ (lines 2, 5, 6), ‘according to’ (line 3) and ‘common’ (line 4).

These represent a justificatory discourse, where conforming to an expected and widely recognised international standard is used as a justification to strengthen China’s positions and arguments and to deflect criticisms. Examples of this are ‘we must conduct fundamental reform in this system so that an investment and funding system that is in line with the international practice and in line with the requirements of a market economy can be put into place’, or ‘the practice of China in the Internet management is also consistent with the established international practice’ as remarked by the premier in response to the journalist’s question on China’s ‘Internet censorship’ issue.

Interestingly, all 6 instances of ‘international practice(s)’ appear in the first two administrations (1998–2012) and mentions of it are conspicuously absent in the latest Xi-Li administration so far (2013–2017). This shows that, at a time when China was less integrated globally and was in a weaker position politically and economically (first two administrations), China tended to use the adherence to international standards as a way to justify what the government had done and proposed to do in English.

Comparative analysis suggests that there are 4 instances of its literal equivalent 国际惯例 in Chinese. Despite the relatively small numbers, this indeed points to a tendency for the interpreters to use this expression and further legitimise the government’s actions in the first two administrations.

Unlike this relatively defensive and conformist mode of discursive articulation, China appears less preoccupied with justifying its actions using the perceived international standards during the Xi-Li administration. Now over three decades into the pragmatist reform, China is increasingly seeking to play a proactive role in shaping the rules of the game as a major global player (e.g., AIIB bank and Belt and Road Initiative). Therefore, the interpreters’ language use both reflects and contributes to the shifting power relations globally against a changing sociopolitical landscape.

*5*.*2*.*2*.2 Glob*. Having explored international*, 全球* and glob* (globe, global, globalised and globalisation) are now investigated in the corpus to study China’s level of global engagement in both subcorpora. As illustrated in Table 4, the interpreters have shown increased alignment through increased (re)production of the related items in each period and overall. Notably, there are considerably more concentrated articulations of the identified items in the third administration.

**Table 4:** Items relating to glob* in both subcorpora and across the administrations.

	Chinese subcorpus (freq/freq per year)	English subcorpus (freq/freq per year)	Increase (percent)
Zhu (1998–2002)	1/0.2 (7082.6 tokens/year)	1/0.2 (6101.6 tokens/year)	0%
Wen (2003–2012)	1/0.1 (8614.5 tokens/year)	18/1.8 (7166.3 tokens/year)	1700%
Li (2013–2017)	14/2.8 (9740.4 tokens/year)	41/8.2 (7698.6 tokens/year)	193%
Overall	16	60	275%

**Fig 4:**
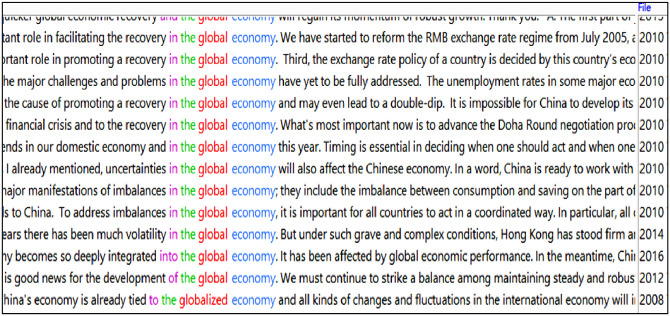
Concordance lines relating to ‘global economy’ and ‘globalised economy’.

A close inspection of the concordance lines containing glob* shows that ‘economy’ is featured prominently in the (re)presentation of China’s discourse on its global engagement. This is evidenced in the following recurring patterns: ‘global economic + noun’ (9 instances), ‘global economy’ (14 instances), ‘global trade’ (7 instances), ‘economic globalisation’ (2 instances), and ‘globalised economy’ (1 instance). Some of the patterns can be found in the concordance lines below (Fig 4). Unsurprisingly, this can partially be explained by the fact that China is itself a big beneficiary from economic reform and economic globalisation. Most of these patterns began to surface around 2008 in the English subcorpus, roughly coinciding with the global financial crisis started that year.

This sudden uptick in the references to global(ised) economy marks a noticeable shift that China’s economy fundamentally transformed from one that was largely inward-looking (focusing on domestic economic policies and initiatives) to one that is inextricably linked to the global economy. Relatively unscathed by the global economic downturn around 2008, China became a major engine for world economic growth and overtook Japan as the world’s second largest economy in the aftermath of the financial crisis. Notably, China’s economic growth started to slow down gradually around 2015, entering into what is officially called a “new normal”. ST-TT comparisons show that the patterns and trends (re)presented in English are accurate reflections of the Chinese originals, hence a general level of interpreter alignment.

**Fig 5:**
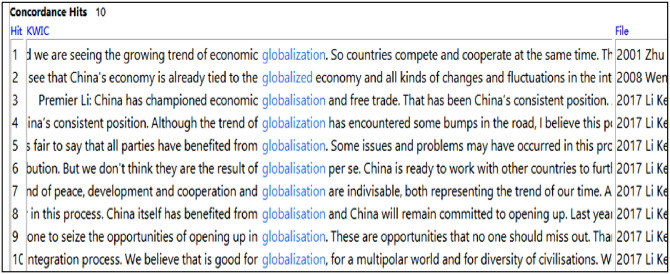
Screenshot of concordance lines featuring globalis* (by year).

Interestingly, for items directly related to globalisation (*globalised* and *globalisation*), diachronically, there is only one explicit mention each in Premier Zhu and Premier Wen’s administrations (in 2001 and 2008), indicating that China in these two periods was still relatively domestically-oriented. Notably, however, there is a sudden spike in 2017 during the Xi-Li period where *globalisation* is mentioned 8 times (Fig 5). At the 2017 press conference, it seems that Beijing became a vocal advocate for globalisation.

To contextualise this, China’s such espousal of globalisation as a vocal proponent in 2017 came at a time when the future of globalisation was shrouded in uncertainty, following the UK’s Brexit Referendum and Donald Trump’s electoral victory. Such concentrated mentions constitute an intertextual response to the recent events that put globalisation potentially in danger, thus reassuring the world of China’s ongoing commitment. As seen in the screenshot, it is constructed discursively that China ‘itself has benefitted from globalisation’ (line 8) and ‘China has championed economic globalisation and free trade’ (line 3), which represent China’s ‘consistent position’ (line 3).

Notably, China’s discourse on its global engagement is also constructed in conjunction with other important concepts: ‘China will remain committed to opening-up’ (lines 8) and ‘seize the opportunities of opening up’ in globalisation (line 9) and ‘peace, development and cooperation and globalisation are indivisible’ (line 7). This suggests the close nexus cognitively between these concepts and China’s general view on various elements central to reform and opening-up. Comparative analyses show that the above patterns are accurate (re)productions of the STs.

## 6. Conclusion and implications

Reform and Opening-up has been an overarching metadiscourse justifying and legitimating China’s sociopolitical systems, development model, and numerous decisions and positions in the entire post-1978 China. The ROU metadiscourse also represents a vital developing discourse for China—the largest developing nation in the world. This study dissected the government-affiliated interpreters’ mediation of the neutral/positive international engagement and global involvement discourses. Bearing the nature of the study in mind, an innovative corpus-based PDA approach was adopted for more systematic and objective analysis. The analysis firstly established the interpreters’ general strengthening of major themes/discourses (e.g. DEVELOPMENT, ECONOMY, REFORM, MARKET, MODERNISATION, STABILITY, OPENNESS, HARMONY, SOCIALISM) through the (over)production of relevant lexical items in English. The interpreters’ proliferated mentions of these lexical items represent a salient case of repetition [6, 23], thus rendering the broader ROU metadiscourse as a whole significantly more emphatic in English. Such overall level comparisons would have been impossible without using a corpus-based approach. From a product-oriented perspective, the interpreters have (re)constructed an even more positive image of the government, for example, being reform-oriented, open, keen on (economic) development and eager to modernise the country in English overall.

With this as a general starting point, the interpreters’ mediation of China’s global involvement and international engagement discourses was analysed at different levels. It is found that at a lexical level China’s global involvement and international engagement discourses have been rendered more emphatic and visible in English overall and also in each administration. Also, diachronically, across the three administrations, facilitated by the interpreters, a positive image of China being internationally engaged and global-minded has been progressively (re)created. At a collocational level, interesting patterns were identified, cognitively revealing China’s thinking patterns and priorities (e.g. ‘global economy’, ‘economic globalisation’, ‘peace, development and cooperation and globalisation’) in its global engagement and integration over the 20 years. These represent an accurate (re)presentation of the Chinese discourses facilitated by those interpreters. Notably, interpreters have tended to repeatedly add salient patterns such as ‘international community’ and ‘international practice(s)’ in English. It is found that China’s discursive pattern has shifted from a defensive, conformist, and justificatory type of discourse in earlier administration(s) towards one that increasingly features initiative, international engagement and recently international leadership as a defender of free trade and champion of an open globalised market (unlike the US under Trump). Doubtlessly, the government-affiliated interpreters have successfully facilitated such discursive changes, helping (re)create an increasingly proactive image of China as a global player.

Looking beyond the traditional conceptualisation of interpreters as agentless and transparent conduits, the press conference interpreters’ strengthening of China’s ROU metadiscourse in general and more specifically China’s global engagement and international involvement discourses points to their key role as interested participants in (re)telling China’s story and (re)articulating sociopolitical truth and knowledge beyond China’s physical borders. Given the outward-facing and high-profile nature of the discursive event, the often more emphatic, forceful and convincing rendering of Beijing’s discourses in English might have far-reaching global ramifications beyond the confines of the press conference hall. That is, the interpreter-mediated discourses can contribute discursively to the constantly shifting and delicate power differentials between China and the more dominant and vociferous West and counterbalance the naturalised and often taken-for-granted Western narratives [39, 40]. Therefore, interpreting in this institutional context can be seen as of strategic importance for China’s development as the second largest economy and the largest developing country in the global south [6, 9], especially given the potentially far-reaching discursive impact of the interpreter-mediated discourse in the global arena [43]. Such interpreter mediation is of particular interest, considering that those interpreters tend to further progress in their careers as government officials, ambassadors, high-profile political commentators and China’s foreign ministers.

Framed within the recent trends of interdisciplinary and digital humanities research, this innovative corpus-based PDA study highlights the vital role of interpreters as indispensable (re)tellers of China’s reform and opening-up discourse in an increasingly mediat(is)ed and (re)mediat(is)ed world. The study ultimately illustrates how a major non-Western developing country from the global South communicates its positive development discourse bilingually in front of the international community. Moving beyond the exploration of unique features of a particular type of language use or genre and the identification of linguistic patterns for pedagogical or lexicographical purposes, this study has demonstrated how a corpus linguistics approach can be fruitfully adopted to help us better understand our world from a broader social, diplomatic and geopolitical perspective.

This interdisciplinary study promises to contribute to image studies, interpreting studies, corpus linguistics, discourse analysis, the political sciences, development studies, and media and communication studies. This empirical study also exemplifies a much-needed ‘outward turn’ in interpreting studies that foregrounds the active sociopolitical and historical shaping role of interpreting and promotes interdisciplinary and mutually enhancing dialogues with other disciplines and areas [43–48]. It is believed that only in this way IS can move beyond the traditional taken-for-granted foci on the ideas of equivalence, faithfulness, invisibility, interpreting quality as well as the long-standing preoccupations with the various internal features, processes, and mechanisms of interpreting as a semi-closed system from within. As for limitations, it is worth noting that the research findings of this article are only true based on the 20 years of corpus data (1998–2017). Since China’s development mentality and international engagement strategies might change in a constantly changing world, China’s discursive articulations in Chinese and the interpreter-mediated discourse in English too are subject to change. Also, the research findings of this study need to be looked at in conjunction with China’s discursive articulations in other discursive events (e.g. foreign ministry spokesperson and foreign minister’s remarks) for a more holistic and comprehensive understanding. The data and topic might also be investigated in a more systematic way using a stratified and layered framework as proposed by Gu [49] from different entry points, combining corpus linguistics and discourse analysis. Also, as an avenue of future research, a text mining machine learning approach, for example, might be applied going forward for a more in-depth analysis of the (diachronic) evolution of the discourses of China, which promises to add new perspectives to this broader topic.

## Supporting information

S1 File (DOC)
