# Supplementary material for: ‘Climate change concerns human survival…and justice in our international community’: A corpus-based positive discourse analysis (PDA) of the largest developing nation’s global involve/engagement discourses (re)told in interpreting
Source: PLoS One. 2023 Apr 20;18(4):e0277705. doi: 10.1371/journal.pone.0277705 (PMC10118079; doi:10.1371/journal.pone.0277705)
Supplement: S1 File — (DOC) [file pone.0277705.s001.doc]

**Short bio:**

With a PhD in Interpreting Studies (University of Manchester) and MA in Conference Interpreting and Translation Studies (University of Leeds) under belt, **Dr Chonglong Gu** is currently assistant professor in translation and interpreting with the Hong Kong Polytechnic University. Previously, he was lecturer/assistant professor in Chinese Translation Studies and programme director of MA in Chinese-English Translation and Interpreting at the University of Liverpool. Chonglong has also taught translation and interpreting in various UK universities including the University of Manchester, University of Leicester, Westminster University, and London Metropolitan University. His recent academic writings have appeared in several SSCI-indexed journals including Target, The Translator, Perspectives, Translation and Interpreting Studies, Discourse, Context and Media, Critical Discourse Studies, Language and Intercultural Communication and various book chapters (Routledge, Springer, Peter Lang etc.). He has co-edited a special issue with New Voices in Translation studies and is co-editor of the Routledge book entitled Translation as a Set of Frames. He has also frequently reviewed SSCI/A&HCI listed journals and book proposals (e.g. Routledge and Cambridge University Press). He is currently the associate editor of IJCETI (The International Journal of Chinese and English Translation & Interpreting) with Prof Binhua Wang (University of Leeds) being the EiC.
